# Who deserves what and why during the COVID‐19 pandemic: Applying the CARIN principles of deservingness to the American welfare state

**DOI:** 10.1111/spol.12859

**Published:** 2022-09-06

**Authors:** David I. Crabtree, Wesley W. Wehde

**Affiliations:** ^1^ Political Science University of Chicago Chicago Illinois USA; ^2^ Political Science Texas Tech University Lubbock Texas USA

**Keywords:** CARIN deservingness principles, COVID‐19 pandemic, social construction, survey experiment, US welfare policy

## Abstract

How does the public decide who is deserving of welfare benefits? To shed light on this question, we investigate whether the CARIN principles of deservingness—specifically the ideas of control, attitude, reciprocity, identity, and need—impact the public's perception of American welfare target groups. We draw contrast between traditional welfare programs and pandemic‐related programs to gain a more comparative understanding of the principles' effects as well as to determine what role the pandemic may play in shaping welfare perceptions. We report that positive, deserving social constructions exist for recipients of both traditional and pandemic‐related welfare programs, and we find evidence that the distinction between traditional and pandemic‐related programs is important for deservingness perceptions in the US. Overall, these results suggest the importance of the CARIN criteria in an American context.

## 1 THE SOCIAL CONSTRUCTION OF DESERVINGNESS AND ITS IMPACT ON POLITICS

Who deserves what and why? In determining how the public answers this question, early scholars emphasised the socially constructed nature of target populations (Schneider & Ingram, 1993). These theories have since spurred waves of both empirical assessment (Al‐Kohlani & Campbell, 2016; Kreitzer & Smith, 2018; Pierce et al., 2014) and practical application to politics (Bell, 2020; Moon, 2020; Schroedel & Jordan, 1998). One specific domain that political scientists and public policy scholars have applied this work to is that of the welfare state (Hussey & Pearson‐Merkowitz, 2012). Benefit recipients of welfare programs can be understood as constituting socially constructed target groups (Kreitzer & Smith, 2018, 769–770), and often these groups differ in their social construction along salient identities like race or citizenship status—both explored to some degree in the US by the current literature (Gilens, 1995; Wright, 1977).

However, missing from the contemporary discussion are the specific criteria through which the American public builds its social construction of welfare target groups. Specifically, how does the public determine whether welfare beneficiaries receive positive or negative social constructions? In addition, of novel interest, the COVID‐19 pandemic introduced a new potential cleavage through which the public may judge welfare target groups: whether or not the group is receiving benefits from a program enacted specifically because of the pandemic.

What concepts are used to socially construct welfare target groups in the US? Are these groups constructed as deserving or undeserving? Do recipients of welfare programs related to the pandemic receive relatively more deserving social constructions than recipients of other, traditional welfare programs? Despite the importance of such questions to policy scholars, they have been left largely unanswered. In this timely study, we examine the social construction of the target groups for traditional welfare and pandemic‐related welfare policy according to the five CARIN principles of deservingness laid out by van Oorschot (2000) and developed by subsequent research in European democracies (Hilmar, 2019; Jørgensen & Thomsen, 2016; Osipovič, 2015). To do so, we draw on a nationally representative survey experiment in the US conducted between April 28th and June 5th in 2021.

Our study both applies the CARIN framework in a novel way to the American context and finds evidence that the principles play a significant role in predicting policy support. Difference of means tests also suggest that the type of welfare benefits received makes a difference in how the public socially constructs the beneficiaries. These results further the field of policy analysis by underscoring the CARIN framework's relevance in constructing American target groups specifically. Finally, our work opens new questions surrounding the importance of pandemics in shaping the way that welfare groups are socially constructed. The study's results are consistent with the theory that external economic shocks (like pandemics) impact the perceived deservingness of welfare beneficiaries because they are not perceived at fault for their economic plight.

We begin with an overview of the literature on socially constructed target groups, subsequent scholarly analysis of the importance of deservingness to politics, and the role that largely European research into the five CARIN principles has played in informing our work. We conclude the literature review and theory section by presenting our primary argument: the pandemic's external, ubiquitous, and far‐reaching economic shock resulted in a supportive shift in the social construction of welfare recipients, and this is best captured by the CARIN principles framework. The introduction of welfare specifically for the pandemic led to the creation of a new, highly deserving target group due to the broad and inclusive nature of benefits like, for instance, stimulus checks. In the methods section, we detail our experimental design. Then, we present our findings and connect them back to our earlier theory. We conclude with the limitations of our work and the questions posed to future researchers analysing the American welfare state.

## 2 LITERATURE REVIEW

### 2.1 Social construction of target populations

Schneider and Ingram's (1993, 1997) seminal writings argue that the social construction of target groups is a variable impacting politics. Their theory centres on the public's normative evaluation of a specific target group and the consequences that this has for the group's experience with government policy (1993, 334). Specifically, target groups that receive positive social constructions are those often stereotyped as ‘deserving, intelligent, honest, public spirited, and so forth’ (1993, 335), and these groups tend to benefit from the policy agenda and its legitimization (1993, 337). Groups with negative social constructions, on the other hand, are often characterised as being undeserving, unintelligent, dishonest, and so on, and they are often subsequently punished by policy (1993, 337). Moreover, they are theorised to have a lower participation rate in political processes (1993, 343–344; Ingram et al., 2007, 106).

Because the social construction of target populations impacts politics through potential participatory effects, it is important for political scientists and policy scholars to consider whether other aspects of politics—like policy support—are impacted. Before evaluating a specific target group, however, it is important to explore what constitutes a positive and negative social construction.

### 2.2 Deservingness perceptions: Operationalising social construction

Since the propagation of Schneider and Ingram's framework, subsequent works have sought to apply the assumptions and predictions of the theory to a wide array of policy domains (Pierce et al., 2014; Ingram et al., 2007, 114–117). In doing so, it has been necessary for researchers to operationalise the idea of ‘social construction’ via a more measurable criterion: deservingness perceptions. Deservingness describes the extent to which particular target groups are worthy of the benefits they receive (or do not receive) (Ingram et al., 2007, 98; Schneider & Ingram, 2005, 1–2).

Research conducted in the field of political psychology found support for the existence of a deservingness *heuristic* which underlies and motivates public support for social policy (Petersen, 2015; Petersen et al., 2011). Such claims underscore the importance of deservingness cues in forming social constructions. Moreover, as Jensen and Petersen (2017) observe, these deservingness heuristics—due to their near‐ubiquitous effects—can have a rather substantial impact on public support for specific social policies. Thus, deservingness perceptions operationalise the broader notion of social constructions by providing an estimate of which groups should receive which benefits.

### 2.3 The CARIN deservingness principles

While prior research (mentioned above) into the social construction of target groups in the US has inspected deservingness broadly, work by European sociologists and political scientists has begun to explore the particular makeup of relevant deservingness cues. Notably, van Oorschot (2000) proposed and argued for the prominence of the five ‘CARIN deservingness criteria’ (van Oorschot & Roosma, 2017). Specifically, these criteria are conceptualised as the principles of control, attitude, reciprocity, identity, and need.

Control is a measurement of how much or little personal blame a group is perceived to have for their circumstances. Attitude represents a target group's perceived gratefulness to society for social benefits. Reciprocity describes the extent to which the target group is perceived to give back to society. Identity is a measurement of a target group's perceived proximity or relatedness to one's own social grouping. Finally, need describes the degree to which a target group is perceived to actually require or truly depend on social benefits (van Oorschot, 2000, 36; van Oorschot, 2006, 26).

Each principle can be visualised on a spectrum, with higher or lower ratings corresponding with greater or lesser deservingness. For example, an archetypically positive, deserving target group would be one which is perceived to be: not in control of their circumstances, grateful to society for their benefits, reciprocating the benefits they are receiving, similar in their relevant or salient identities to the rest of society, and truly in need of the benefits they are receiving (van Oorschot, 2000, 36). Furthermore, it logically follows that an archetypically negative and undeserving target group would have the opposite ratings of the aforementioned principles.

Meuleman et al. (2020) argued that these criteria serve as a mediator between personal characteristics and policy support, and their work has illustrated construct validity for each of the five principles. Furthermore, this framework has been applied in a variety of European contexts (Hilmar, 2019; Jørgensen & Thomsen, 2016; Osipovič, 2015), but—to our knowledge—it has not yet been extensively applied to measure the deservingness of welfare target groups in the US (for one example, see Jensen & Petersen, 2017). It is worth noting that there has been no shortage of research related to the pandemic's impact on, for instance, risk perceptions (Breznau, 2021), social behaviour (Erev et al., 2020), and health spending (Busemeyer, 2021). Nonetheless, there is a need for work applying the CARIN framework in the US.

### 2.4 Welfare target groups in the United States: A comparative case during the COVID‐19 pandemic

Recently, research into the COVID‐19 pandemic's impact on targets of western democratic welfare states has proliferated. Examples include studies examining: perceptions of the unemployed in Australia (Suomi et al., 2020), deservingness perceptions and cash transfer support for target groups in Canada (Bridgman et al., 2021), and support among the German public for medical solidarity and financial redistribution (Koos & Leuffen, 2020). A common observation in the former two of the three studies mentioned is that the pandemic, as an independent variable, may be impacting various public perceptions of target groups' control over their economic circumstances. This finding, in line with van Oorschot (2006, 26), corresponds with greater deservingness perceptions for welfare target groups in times of high unemployment, specifically, as individuals are not viewed at fault for the external economic shock of the pandemic. In other words, perhaps the control rating of target groups is perceived in a deserving light. Such a hypothesis would explain the more ‘universalist’ deservingness ratings reported in Australia (Suomi et al., 2020) and Canada (Bridgman et al., 2021).

Largely overlooked in this global discussion is the distinction between welfare programs enacted not merely ‘in pandemic times’ (as has been explored), but rather enacted specifically *for* pandemic circumstances. This latter type of welfare—with its own target group—is referred to in this study as ‘pandemic‐related welfare’. It includes benefits enacted specifically in the context of the pandemic like direct payments (i.e. stimulus checks), grants or forgivable loans, special unemployment assistance, payroll tax credits for businesses, student loan relief, eviction relief, food assistance, and more (see, Benefits.gov 2021). This study contrasts pandemic‐related welfare with ‘traditional welfare’ which consists of any welfare program enacted for reasons unrelated to the pandemic, such as SNAP, TANF, Medicaid, SSI, and so on.

Also understated in current literature is the importance of appreciating the social construction of welfare target groups in the US in comparative context. While, as mentioned, some western democracies have exhibited increased universalism in public deservingness perceptions (Bridgman et al., 2021; Suomi et al., 2020), this has not been the case in all instances. In the aforementioned study in Germany (Koos & Leuffen, 2020), citizens demonstrated rather durable conditionality in forming their perceptions. Furthermore, while it is plausible that the pandemic is responsible for the purported attitude shifts in Canada and Australia, Bridgman et al. (2021) note in their discussion that the importance of some deservingness criteria may have simply changed as a function of time instead. Thus, open debate remains as to how relevant the pandemic is in the public's formation of deservingness perceptions.

Drawing on van Oorschot's (2000 and 2006) descriptions of the CARIN principles, we can logically infer the effect of the pandemic on perceptions of welfare recipients in the United States. The pandemic's economic shock forced declines in consumer spending, raised unemployment rates, and placed vulnerable socioeconomic groups at higher risk of poverty (Bauer et al., 2020). Therefore, recipients of traditional programs (such as SNAP, TANF, medicaid, SSI, and so on) may be viewed as in less *control* of their financial circumstances, because a force other than themselves is responsible for their dire economic condition. Furthermore, the pandemic's economic impact may heighten public perception of recipients' true need of program benefits, because the pandemic's economic consequences have made subsistence harder. By contrast, we do not expect for the pandemic to alter the perception of whether traditional recipients give back to society (reciprocity), are similar in identity to the public, or are grateful for their benefits (attitude). Because the pandemic‐related welfare target group is comparatively more inclusive and ubiquitous than the traditional welfare target group, we expect for the former to benefit from positive ratings of all principles, because anyone receiving—for instance—stimulus checks, will be seen as not to blame for receiving the benefit, grateful to receive the benefit, reciprocating through their taxes, and similar in identity to the rest of society.

### 2.5 Hypotheses

To address the current shortcomings in the extant literature, we proposed the following hypotheses^1^ which this study will test:

H1—We expect recipients of traditional welfare to have positive ratings for the principles of control and need and negative ratings for the principles of attitude, reciprocity, and identity. By contrast, we expect recipients of pandemic‐related welfare to have positive ratings for control, attitude, reciprocity, and identity.

H2—We expect recipients of pandemic‐related welfare programs in the US to be socially constructed fundamentally more deservingly than recipients of traditional welfare programs such that:

- Recipients of pandemic‐related welfare are perceived to have less control over their financial circumstances.
- Recipients of pandemic‐related welfare are perceived to have higher ratings of the attitude principle.
- Recipients of pandemic‐related welfare are perceived to have higher ratings of the reciprocity principle.
- Recipients of pandemic‐related welfare are perceived to be more similar in identity to respondents.

H3—We expect positive ratings of each of the principles to be positively correlated with welfare policy support. This would suggest that respondents who perceive welfare recipients to be: not in control of their economic circumstances, grateful for their benefits, reciprocating their benefits, similar in identity to the respondent, and genuinely in need of their benefits will thus support policy that benefits these recipients. Specifically, we expect for the control and identity principles to have the strongest positive correlation with policy support.

## 3 DATA AND METHODS

### 3.1 Survey instrument

The sample (N = 1650) for this study was gathered via a survey experiment active from April 28 to June 5, 2021 in the United States. The survey measured respondents' demographics, welfare attitudes, and perceptions of welfare recipients in that order. Respondents are nationally representative of the US population on qualities of age, sex, and ethnicity. Before providing the relevant data, respondents were given an attention check question which established a definition of pandemic‐related welfare programs and traditional welfare programs. Our study's sample only includes data from respondents who passed this attention check.

### 3.2 Experimental design

For this section of the survey, respondents were randomly divided into two groups. The first group (n = 823) received a set of questions asking them to rate their perceptions of traditional welfare recipients along the five CARIN deservingness principles (van Oorschot, 2000). The second group (n = 827) received a similarly worded set asking them to rate their perceptions of pandemic‐related welfare recipients along four of the principles: control, attitude, reciprocity, and identity. Measurement for the need principle rating of pandemic‐related welfare recipients was not available for this survey because the traditional welfare need rating was inadvertently collected twice (see Table 1 which shows only one measurement for the need principle, where n = 1650). Respondents for the first group did *not* receive the questions shown to the second group and vice versa.

**TABLE 1:** Descriptive statistics of principle ratings

	Pandemic‐related welfare recipients (N = 827)	Traditional welfare recipients (N = 823)	Overall (N = 1650)
Control principle			
Mean (SD)	4.43 (1.31)	3.99 (1.40)	4.21 (1.37)
Median [Min, Max]	5.00 [1.00, 6.00]	4.00 [1.00, 6.00]	4.00 [1.00, 6.00]
Attitude principle (reverse coded)			
Mean (SD)	3.44 (1.57)	3.19 (1.48)	3.31 (1.53)
Median [Min, Max]	3.00 [1.00, 6.00]	3.00 [1.00, 6.00]	3.00 [1.00, 6.00]
Reciprocity principle (reverse coded)			
Mean (SD)	3.36 (1.45)	3.10 (1.39)	3.23 (1.43)
Median [Min, Max]	3.00 [1.00, 6.00]	3.00 [1.00, 6.00]	3.00 [1.00, 6.00]
Identity principle			
Mean (SD)	3.92 (1.46)	3.40 (1.54)	3.66 (1.52)
Median [Min, Max]	4.00 [1.00, 6.00]	3.00 [1.00, 6.00]	4.00 [1.00, 6.00]
Need principle^a^			
Mean (SD)		4.35 (1.28)	
Median [Min, Max]		5.00 [1.00, 6.00]	

^a^ Mean for need principle of traditional welfare recipients has n = 1650, as the same treatment was inadvertently applied to all survey respondents for the need question.

### 3.3 Measuring the deservingness principles: Experimental design

In operationalising the CARIN criteria for this survey, we drew on previous applications of the concepts by Meuleman et al. (2020) as well as van Oorschot (2000). Below, we list the deservingness principle in italics as well as the wording of the question used to measure it. Respondents only saw the prompt and were not given context as to the concepts (in italics) being operationalised.

*Control*—In general, most benefit recipients of [1. traditional welfare / 2. pandemic‐related welfare] programs are not to blame for their financial circumstances.

*Attitude*—In general, most benefit recipients of [1. traditional welfare / 2. pandemic‐related welfare] programs show little gratitude to society.

*Reciprocity*—In general, most benefit recipients of [1. traditional welfare / 2. pandemic‐related welfare] programs do not contribute to such collective programs themselves.

*Identity*—In general, I identify more with recipients of [1. traditional welfare / 2. pandemic‐related welfare] programs than with individuals who receive benefits from [1. welfare programs that were initiated in response to the ongoing pandemic / 2. traditional welfare programs].

*Need*—In general, benefit recipients of traditional welfare programs are in strong need of the benefits they receive.

This survey utilised 6‐point scales ranging from ‘strongly disagree’ to ‘strongly agree’, and these did not include neutral ‘midpoint’ values. Contemporary debate surrounds the issue of midpoints in survey design, which merits brief exposition here due to its impact on the interpretation of results. Some scholars (see Wang & Krosnick, 2020 as well as O'Muircheartaigh et al., 2000) argue that midpoint inclusion reduces random measurement error while maintaining validity. By contrast, others find that midpoint inclusion biases responses towards the ‘neutral’ category even when such responses are not accurate (see Chyung et al., 2017; Weems & Onwuegbuzie, 2001). We concur with this latter assessment of available evidence, and we contend that the inclusion of midpoints in our study in particular would have been problematic due to the socially sensitive nature of our questions encouraging response bias.

For all analyses, the attitude and reciprocity measures are reverse coded such that higher levels suggest greater deservingness in line with the three other principles. Thus, in all cases, higher scores indicate higher deservingness. This design provides an estimate of how the public builds its social construction of both traditional welfare recipients and pandemic‐related welfare recipients according to the CARIN principles, with scores of 3 and below indicating undeserving ratings and scores of 4 and higher indicating deserving ratings. Table 1 shows the distribution of responses per prompt received.

### 3.4 Dependent variables

*Principle ratings*—The CARIN principles described above serve as dependent variables in the first and second sets of tests of this study. Specifically, we first test the provided principle ratings for significant positive or negative valence (deservingness). Second, we test whether the welfare target group presented makes a difference in the positive or negative valence of the given principle rating.

*Support for pandemic‐related welfare policy*—Before reaching the experimental section of the survey involving principle ratings, all respondents provided measurements of their current attitude towards pandemic‐related welfare policy. Specifically, respondents were provided a descriptive summary of the CARES Act and were then asked to rank their level of support or opposition to policy that perpetuates pandemic‐related welfare programs. The responses for this question ranged on a 6‐point scale from ‘strongly oppose’ to ‘strongly support’.

### 3.5 Independent variables

*Prompt received*—As seen in the description of the experimental design, respondents were shown one of two sets of prompts in order to provide principle ratings for both traditional welfare recipients and pandemic‐related welfare recipients. Because of this, the type of prompt received serves as an experimental treatment impacting the responses for principle ratings.

*Principle ratings*—In the third set of tests for this study, the principle ratings serve as an independent variable to determine the relationship of positive or negative valence with welfare policy support.

*Demographics*—A small set of demographic variables serve as covariates in one of the models for welfare policy support. Specifically, demographic measurements include age (continuous), gender (a dichotomous measurement where 1 = male and 0 = female), income factored into quintiles where each quintile above the first is compared with the first's effects, ethnicity (a dichotomous measurement where 1 = white and 0 = non‐white), education (where 1 = bachelor's degree or higher and 0 = below a bachelor's degree), and ideology (7‐point scale). Table 2 illustrates descriptive statistics for the policy support variable and for demographics.

**TABLE 2:** Descriptive statistics of policy support and demographics

				Overall (N = 1650)
Variable	Mean	SD	Median	[Min, Max]
Support for pandemic‐related welfare policy	4.59	1.44	5	[1, 6]
Age	45.4	17.6	44	[18, 93]
Male	0.487	0.5	0	[0, 1]
Income quintiles	2.68	1.42	2	[1, 5]
White	0.748	0.434	1	[0, 1]
Bachelor's degree and higher	0.440	0.497	0	[0, 1]
Ideology	3.92	1.83	4	[1, 7]

### 3.6 Preliminary correlation matrix

Table 3's bivariate correlations reveal the underlying relationship between the CARIN principle measures we used, similar to Meuleman et al. (2020). It suggests that all of the CARIN principles are significantly correlated with one another. We also include Table A1 in the Appendix which compares the correlation coefficients for the deservingness principles across both treatments; we find similar correlation magnitudes across both treatments, providing suggestive evidence that the structure of these beliefs is consistent across policies. In general, our findings replicate those of the Meuleman et al. (2020) study in Belgium with a few key exceptions. Importantly, while all of our correlations are statistically significant, none are particularly large, suggesting these measures are capturing distinct underlying principles of deservingness. Specifically, unlike the previous study, we find a negative correlation between identity deservingness and attitude and reciprocity deservingness (after the recoding). This suggests respondents who perceive welfare recipients, both pandemic‐related and traditional, as more deserving due to their shared identity viewed those same welfare recipients as less likely to show gratitude for those programs and less likely to contribute to the programs, with the type of welfare program held constant.

**TABLE 3:** Correlation (Pearson's) matrix of independent and dependent variables

	COVID policy support	Control principle	Attitude principle	Reciprocity principle	Identity principle	Need principle	Age	Male	Household income (quintiles)	White	Bachelor's degree
Control	0.38***										
Attitude	0.23***	**0.14** ***									
Reciprocity	0.13***	**0.09** ***	**0.58** ***								
Identity	0.15***	**0.37** ***	**−0.13** ***	**−0.14** ***							
Need	0.43***	**0.57** ***	**0.14** ***	**0.10** ***	**0.29** ***						
Age	0.00	−0.04	0.08**	0.06*	−0.17***	−0.04					
Male	−0.04	0.02	−0.08***	−0.06*	0.11***	0.01	−0.04				
HH income	−0.05*	0.03	−0.14***	−0.14***	0.05*	0.01	0.09***	0.21***			
White	0.03	0.05	−0.09***	−0.05*	−0.01	0.00	0.26***	0.04	0.19***		
Bachelor's	−0.02	0.08***	−0.06*	−0.08***	0.06*	0.08**	0.08***	0.18***	0.45***	0.10***	
Ideology	−0.21***	−0.14***	−0.14***	−0.15***	−0.07**	−0.19***	0.27***	0.01	0.01	0.16***	−0.02

*Note*: The boldface values in this table represent the correlation between any two CARIN Principles.

*** *p* < 0.001; **p < 0.01; *p < 0.05.

## 4 RESULTS

### 4.1 Testing the positive and negative valence of social constructions

**FIGURE 1:**
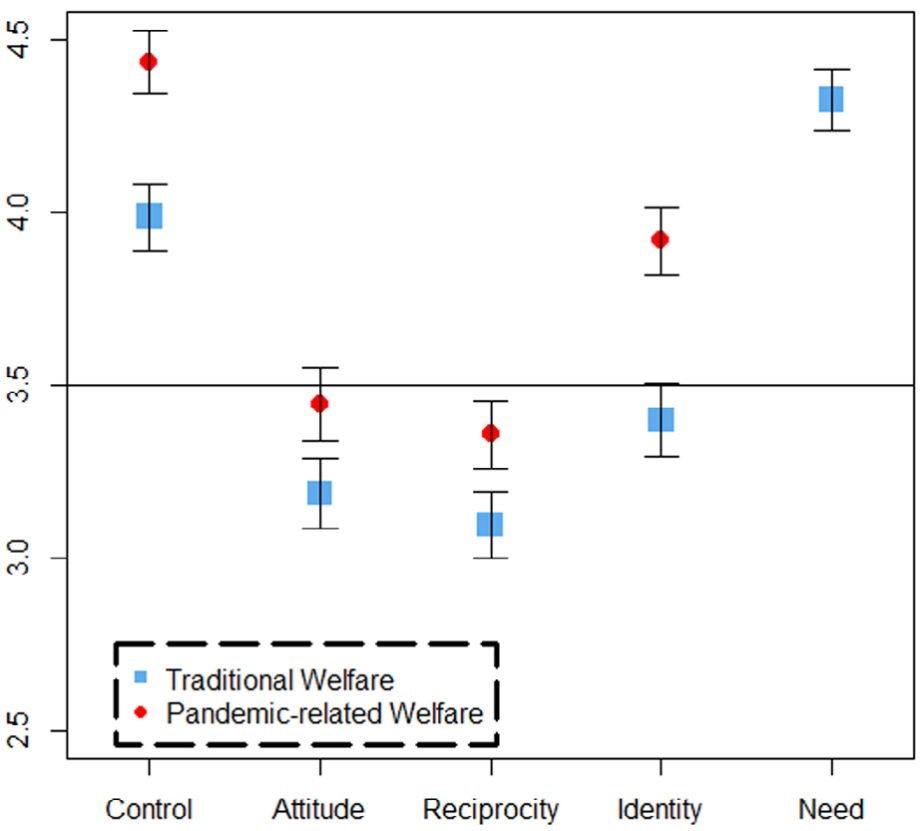
Means of CARIN principle rating [Colour figure can be viewed at wileyonlinelibrary.com]

We begin by examining the extent of positive or negative valence—or deservingness—in the social construction of traditional welfare recipients and pandemic‐related welfare recipients, respectively. To do so, we use one‐sample, one‐sided *t*‐tests to determine whether the mean of each of the available principles is significantly higher or lower than ‘neutral’ on a 6‐point scale. Because no ‘neutral’ response was included in our survey (see Methods section), the logical cutoff for determining a neutral score lies in‐between 3 (slightly disagree) and 4 (slightly agree). Therefore, a mean significantly higher or lower than 3.5 will indicate that the particular welfare target group has a positive or negative valence, respectively, for the specific principle tested. A mean not significantly different from 3.5 will indicate a neutral valence. Figure 1 illustrates the sample means for each of the principle ratings collected per prompt along with 95% confidence intervals.

#### 4.1.1 Traditional welfare recipients

For survey respondents who received the set of prompts concerning traditional welfare recipients, the control and need ratings were found to have means of 3.99 and 4.33, respectively. Because the means were found to be significantly higher than 3.5 (*t* = 10 and 18.2, *p*‐values < 0.01), they contribute positively to the social construction of traditional welfare recipients. Specifically, they indicate that traditional welfare recipients were perceived: (1) to have little control over their financial circumstances and (2) to be in need of the benefits they receive. This finding supports hypothesis H1, which predicted traditional welfare recipients would be perceived to be truly not in control of their financial circumstances and in real need of assistance in the context of the pandemic's economic consequences.

Conversely, negative valence was concluded from the attitude and reciprocity ratings. This is because their means (3.19 and 3.10, respectively) are significantly lower than the predicted neutral value of 3.5 (*t* = −6.1 and −8.3, *p*‐values < 0.01). In addition, the identity rating was also found to have negative valence. The mean rating (3.40) was less than 3.5 (95% confidence interval surrounding negative infinity and 3.4867, *t* = −1.9, *p*‐value < 0.05). Thus, traditional welfare recipients were perceived: (1) to show little gratitude to society, (2) to contribute little to collective benefit programs, and (3) to not be similar in identity to respondents, on average. These findings further support H1, and they are consistent with the theory that the pandemic's economic consequences have *not* led the public to create positive images of traditional welfare recipient's as grateful, reciprocating, or ‘similar to me’.

Ultimately, while some ratings (control and need) indicate a positive social construction for traditional welfare recipients, other ratings (attitude, reciprocity, and identity) confer negative valence. It seems arguable that overall, this target group enjoys a more deserving social construction at the time measured, as prior literature has indicated the relative importance of the control principle in manipulating deservingness perceptions (van Oorschot, 2006, 26). In addition, the positively rated principles have the most extreme means out of all the principles tested.

#### 4.1.2 Pandemic‐related welfare recipients

For pandemic‐related welfare recipients, the mean ratings for the control and identity principles were 4.43 and 3.92, respectively. These both contributed to a positive social construction for this target group because the positively worded prompts coincided with means greater than 3.5 for control and identity (*t* = 20.4 and 8.2, *p*‐values < 0.01). These findings lend further support to H1 which predicts control and identity will exhibit positive valence to the social construction of pandemic‐related welfare recipients.

By contrast, findings for the reciprocity and attitude ratings do not support H1. For reciprocity, its mean (3.36) being lower than 3.5 contributes an undeserving element to the social construction of this target group (*t* = −2.8, *p*‐value < 0.01). The attitude rating exhibited a mean of 3.44, which is indistinguishable from 3.5 (*t* = −1.1, *p*‐value = 0.1463). Thus, the attitude principle contributes neither positively nor negatively to the target group's social construction.

Overall, the control and identity principles contribute to a positive social construction for pandemic‐related welfare recipients, and the ‘strength’ of these ratings (4.43 and 3.92, respectively) seems to outweigh the strength of reciprocity's negative rating (3.36) and attitude's neutral rating (3.44). Therefore, we conclude pandemic‐related welfare recipients have a net‐positive, deserving social construction according to the four CARI principles measured. Thus, we have mixed evidence for the second half of H1, which predicts pandemic‐related welfare recipients to receive strong deservingness perceptions due to the inclusive nature of the programs.

### 4.2 Comparing the principle ratings for traditional and pandemic‐related welfare recipients

To determine if the type of welfare received makes a difference in the public's construction of target groups, we used two‐sample *t*‐tests to see if each group's mean for their respective principle ratings significantly differed from each other. Below, we list the results of these difference of means tests for each available principle:

#### 4.2.1 Control rating

Respondents indicated an average control rating of 4.4 for pandemic‐related welfare recipients and 4.0 for traditional welfare recipients, and we found this difference of means to be significant (*t* = 6.7, *p*‐value < 0.01). This indicates that the target group for pandemic‐related welfare is perceived to be in less control of their financial circumstances, and thus they have a more positive social construction of the control principle. This supports part I of H2.

#### 4.2.2 Attitude rating

Mean attitude ratings were indicated as 3.4 for pandemic‐related welfare recipients and 3.2 for traditional welfare recipients. We also found this difference to be significant (*t* = 3.4, *p*‐value < 0.01). This finding suggests that traditional welfare recipients are perceived to be less grateful than pandemic‐related welfare recipients, and this supports H2 part II.

#### 4.2.3 Reciprocity rating

For pandemic‐related welfare recipients, the average reciprocity rating of 3.4 was found to be statistically different from the traditional welfare recipients' average of 3.1 (*t* = 3.7, *p*‐value < 0.01). Thus, traditional welfare recipients are perceived, on average, to not contribute to collective benefits programs as much as pandemic‐related recipients. This finding supports H2 part III.

#### 4.2.4 Identity rating

The pandemic‐related target group received a mean of 3.9 on the identity rating while the traditional welfare target group's mean was 3.4. This difference was also found to be significant (*t* = 7.0, *p*‐value < 0.01), and it indicates that, on average, individuals identify more with the pandemic‐related welfare target group than the traditional welfare counterpart. This supports H2 part IV. Overall, across all principles measured, the target group for pandemic‐related welfare received more positive ratings, and thus they can be interpreted as being ‘more deserving’ than the traditional welfare target group across these principle measurements.

### 4.3 Examining the relationship between demographics and deservingness principles

We are also interested in how demographics and our experimental treatment are related to deservingness principle ratings. Thus, we include linear (OLS) regression models examining the determinants of each in Table 4 below.

**TABLE 4:** Linear regression: Experimental treatment and demographics on CARIN principles

	Dependent variable				
	Control	Attitude	Reciprocity	Identity	Need^a^
	(1)	(2)	(3)	(4)	(5)
Traditional welfare treatment	−0.449*** (0.068)	−0.285*** (0.075)	−0.283*** (0.071)	−0.521*** (0.075)	
Age	−0.003 (0.002)	0.013*** (0.002)	0.011*** (0.002)	−0.014*** (0.002)	0.0003 (0.002)
Male	0.031 (0.071)	−0.109 (0.078)	−0.050 (0.073)	0.277*** (0.077)	0.032 (0.066)
Income second quintile	−0.096 (0.098)	0.022 (0.108)	−0.020 (0.102)	−0.195* (0.108)	−0.029 (0.092)
Income third quintile	−0.141 (0.107)	−0.040 (0.118)	−0.167 (0.111)	−0.363*** (0.117)	−0.236** (0.100)
Income fourth quintile	−0.099 (0.118)	−0.240* (0.131)	−0.210* (0.123)	−0.043 (0.130)	−0.176 (0.111)
Income fifth quintile	−0.125 (0.120)	−0.566*** (0.132)	−0.519*** (0.125)	0.074 (0.132)	−0.039 (0.113)
White	0.257*** (0.084)	−0.268*** (0.092)	−0.108 (0.087)	0.093 (0.092)	0.090 (0.079)
Bachelor's degree	0.231*** (0.077)	−0.022 (0.085)	−0.096 (0.081)	0.142* (0.085)	0.217*** (0.073)
Ideology	−0.098*** (0.019)	−0.136*** (0.021)	−0.131*** (0.020)	−0.023 (0.021)	−0.129*** (0.018)
Constant	4.735*** (0.128)	3.738*** (0.141)	3.647*** (0.133)	4.517*** (0.140)	4.747*** (0.116)
Observations	1533	1534	1529	1533	1536
*R* ^2^	0.059	0.076	0.068	0.087	0.045
Adjusted *R* ^2^	0.053	0.070	0.062	0.081	0.039

^a^ Need principle was not experimentally manipulated.

* *p* < 0.1; **p < 0.05; ***p < 0.01.

Table 4 demonstrates that across all four examined principles the traditional welfare experimental treatment is associated with lower levels of deservingness. Thus, we have further evidence for H2 that pandemic welfare recipients were more positively socially constructed than traditional welfare recipients, despite the overlap in the recipients of both types of welfare. In addition, Table 4 demonstrates no demographic variable is consistently related to all five principles. Political ideology is associated with lower deservingness scores for four of the five principles but displays no significant relationship with the identity principle.

### 4.4 The relationship between principle ratings and welfare policy support

To provide evidence of the importance of the principles to the policy process, we ran linear regression models using respondents' support for pandemic‐related welfare initiatives as the dependent variable. Table 5 shows the results for three regression models. Model 1 illustrates the relationships between the principle ratings and COVID policy support, while Model 2 incorporates the principle ratings and demographics covariates. Model 3 includes the principle ratings and their interaction with the experimental treatment representing the two target groups (traditional welfare recipients and pandemic‐related welfare recipients). Finally, Model 4 examines the principle ratings, the interaction with the two prompts, and a short list of demographic covariates.

**TABLE 5:** Linear regression: CARIN principles, their interaction w/prompt, and multiple variable model on COVID policy support

	Dependent variable			
	COVID policy support			
	(1)	(2)	(3)	(4)
Traditional welfare treatment			0.799*** (0.287)	0.737** (0.295)
Control rating	0.354*** (0.026)	0.349*** (0.027)	0.364*** (0.036)	0.368*** (0.038)
Attitude rating	0.182*** (0.027)	0.167*** (0.027)	0.175*** (0.037)	0.155*** (0.038)
Reciprocity rating	−0.010 (0.028)	−0.039 (0.029)	0.010 (0.040)	−0.022 (0.041)
Identity rating	0.048** (0.023)	0.041* (0.025)	0.151*** (0.033)	0.133*** (0.035)
Age		0.003 (0.002)		0.002 (0.002)
Male		−0.045 (0.069)		−0.025 (0.069)
Income second quintile		0.215** (0.095)		0.177* (0.095)
Income third quintile		0.009 (0.103)		−0.021 (0.103)
Income Fourth Quintile		−0.138 (0.115)		−0.173 (0.115)
Income fifth quintile		−0.064 (0.117)		−0.105 (0.118)
White		0.188** (0.082)		0.182** (0.082)
Bachelor's degree		−0.103 (0.075)		−0.109 (0.075)
Ideology		−0.125*** (0.019)		−0.124*** (0.019)
Control * Prompt			0.006 (0.052)	−0.015 (0.054)
Attitude * Prompt			0.005 (0.053)	0.020 (0.054)
Reciprocity * Prompt			−0.022 (0.057)	−0.014 (0.057)
Identity * Prompt			−0.196*** (0.048)	−0.177*** (0.050)
Constant	2.359*** (0.139)	2.806*** (0.197)	1.848*** (0.220)	2.374*** (0.257)
Observations	1605	1498	1605	1498
*R* ^2^	0.181	0.214	0.192	0.223
Adjusted *R* ^2^	0.179	0.208	0.187	0.214

* *p* < 0.1; **p < 0.05; ***p < 0.01.

#### 4.4.1 Model 1: The principles

The linear regression output showed significant relationships for the control, attitude, and identity principles with regard to policy support for pandemic‐related welfare programs. The positive relationships for control, identity, and attitude conform with the expectation that social constructions with positive valence will coincide with supportive welfare attitudes. Of the principles tested, the strongest relationship was demonstrated by the control principle, as predicted in H3. Specifically, Model 1 predicts that, on average, a 1‐point increase in deservingness for control results in a 0.356 increase in support for pandemic‐related welfare policy when accounting for the effects of the attitude, reciprocity, and identity principles. The only result unexpected by H3 is the small coefficient for reciprocity, which remains insignificant in all four models.

#### 4.4.2 Model 2: The principles and covariates

Model 2 suggests the key deservingness relationships with COVID‐19 policy support are robust to inclusion of a commonly used set of covariates. Coefficients remain remarkably similar in magnitude with only the coefficient on the identity principle significant only at the 0.01 level and no longer meeting the most commonly used level of significance. These results underscore the predictive power of the CARIN principles over policy support.

#### 4.4.3 Model 3: The principles and their interaction with the prompt received

When accounting for whether respondents received prompts for traditional or pandemic‐related welfare recipients, the model output indicated stronger relationships for the control and identity ratings. Importantly, the interaction between identity and prompt was found to be significant (*p*‐value < 0.01). This should, perhaps, not be surprising as it indicates that while, in general, some respondents may be simply predisposed to identify with either category of welfare recipient, accounting for the particular target group that respondents were assigned provides a better model of the identity principle.

#### 4.4.4 Model 4: The principles, the interaction terms, and demographics

**FIGURE 2:**
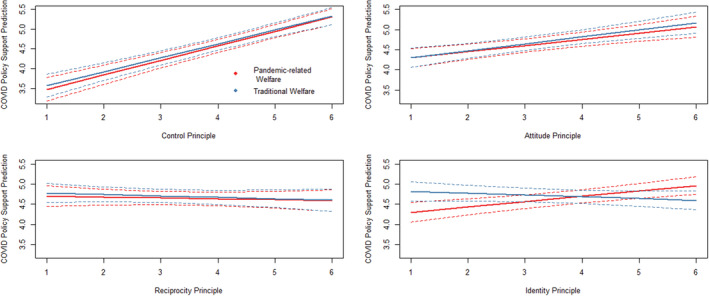
COVID‐19 policy support predictions by deservingness principle and experimental treatment [Colour figure can be viewed at wileyonlinelibrary.com]

The output for Model 4 indicates that, when accounting for the principles and their interaction with the two sets of prompts, the only demographics with a significant relationship were ethnicity (white/non‐white) and ideology. In addition, the Models 3, 4, and Figure 2 suggest that there is no difference in the effect of the principles on policy support *contingent on welfare type* (except for the case of identity), indicating that in most cases the actual interaction between principle and prompt was not significant for impacting policy support. Importantly, though, Table 4 shows that Model 4, specifically, is capable of explaining about 21% of all variance in the dependent variable. Nested *F* tests (*F* = 4.9, *p* < 0.01; *F* = 4.2, *p* < 0.01) reveal that Models 3 and 4, including the interaction term, perform better than their corresponding models without the interaction (Models 1 and 2, respectively). This suggests the important role deservingness principles, and their contingence on the target population, play in predicting COVID‐19 policy support in the US Figure 2 below demonstrates the potential contingent relationships between target populations, deservingness principles, and COVID‐19 policy support.

Figure 2, consistent with Models 3 and 4 in Table 5, suggest that only the identity principle's relationship with COVID‐19 policy support is contingent upon which prompt or treatment respondents received. For respondents who saw the traditional welfare treatment, the relationship between identity and COVID‐19 policy support is negative. On the other hand, the relationship between identity deservingness and COVID‐19 policy support is positive for recipients of pandemic‐related welfare. This suggests that the policy domain structures deservingness perceptions which can ultimately affect policy support for the identity principle. Given the widespread nature of pandemic‐related welfare programs, it may be unsurprising that when prompted this policy domain led to more positive deservingness attitudes for the identity principle. Unlike traditional welfare in the US, which is often contingent upon socio‐economic standing, the pandemic affected everyone and many who do not usually participate in the welfare state did as a result; thus, respondents were better able to identify with recipients, and this led to higher levels of policy support.

## 5 DISCUSSION

We found partial support for H1, which concerned the particular valence of each principle for the two target groups tested. Additional two sample t‐tests and an OLS model fully supported H2, which hypothesized that pandemic‐related welfare recipients would be, relatively speaking, constructed more positively than traditional welfare recipients. Finally, in support of H3, linear regression models indicated that van Oorschot's (2000) principles framework—applied in an American context—is correlated with the public's attitudes towards welfare policy support. Every principle except reciprocity was positively correlated with policy support.

### 5.1 Deriving deserving social constructions from the CARIN principles of deservingness

The deserving ratings for control and need were larger than the undeserving ratings given for attitude, reciprocity, and identity, and prior research (van Oorschot, 2006, 26; Jeene et al., 2014; De Swaan, 1988) as well as this study's linear regression models on policy support suggest that the control principle is the most important factor in the public's formation of deservingness perceptions. Therefore, benefit recipients of traditional welfare programs may, at present, be largely constructed as deserving of welfare benefits due to their perceived lack of control over their financial circumstances and genuine need for help.

The second half of H1 (predicting deserving ratings of all principles for pandemic‐related recipients) was only partially supported, with the target group receiving positive, deserving ratings for the control and identity principle but not reciprocity (undeserving) or attitude (neutral). However, much larger means and t‐statistics for control and identity indicate that recipients of pandemic‐related welfare likely possess a deserving social construction in present circumstances. It must be noted, however, that the public may be less sanguine as to the group's gratefulness or willingness to reciprocate benefits according to the results for the attitude and reciprocity principles.

In addition, our research generally confirms, in the US, the findings from other studies that use the CARIN principles in contexts other than the US. The correlation structures between principles are comparatively similar with the exception of the relationship between the identity principle and the attitude and reciprocity principles. In our data, these correlations are negative instead of positive. We believe this may be because we collected data in two specific policy contexts as opposed to the more general construction of previous work (Meuleman et al., 2020). We ultimately conclude that the five CARIN principles constitute conceptually distinct measurements of deservingness for the purpose of our study in the context of American welfare perceptions, although we do not claim this is true in every case.

### 5.2 Contextualising the COVID‐19 pandemic's effect on deservingness perceptions

In concurrence with the findings of Suomi et al. (2020) in Australia as well as those of Bridgman et al. (2021) in Canada, we find that welfare recipients in the United States are perceived as having little control over their current economic circumstances. This is particularly true for benefit recipients of programs targeted specifically at providing economic relief from the pandemic. It should come as no surprise that COVID‐19 has had such an impact on perceptions of control: analysts note that the job‐losses related to the pandemic erased 113 consecutive months of job growth in the US, and 2020 saw the largest month‐to‐month decline in consumer spending since the US Census Bureau began monitoring such data (Bauer et al., 2020).

Interestingly, these effects seem to be even more salient to the public when asked to provide a control rating for recipients of pandemic‐related welfare policy (as opposed to traditional) even though both target groups are affected by the economic consequences of the pandemic. As we state in our literature review, we believe this to be because there is a conceptual difference between welfare *during the pandemic* and welfare *for the pandemic*, which prior work has failed to fully appreciate. While both target groups have the benefit of deserving control ratings, pandemic‐related welfare recipients have the comparative advantage of receiving a broad, inclusive program, which we hypothesize heightens respondents' perception of recipients' gratefulness, reciprocity, relatedness (identity) and, again, their vindication of recipients' economic plight (control). In our results, the pandemic‐related target group received more deserving ratings along all four principles, consistent with our theory.

### 5.3 Consequences for politics and the importance of the CARIN framework

There is good reason to believe that these differences will have political consequences. Positive social perceptions—especially whether or not the group is perceived as responsible for their circumstances or similar in identity to the public (van Oorschot, 2000, 43)—are associated with greater deservingness for welfare benefits (Petersen et al., 2011). This, in turn, is theorised to lead to supportive changes through the democratic policy process (Schneider & Ingram, 1993, 337). Table 5 in our own results further support this literature, documenting the positive relationship between deserving principle ratings and support for welfare related to COVID‐19 in the US. Thus, for as long as the pandemic (and a target group for those affected by it) exists, these high deservingness perceptions for relevant benefit recipients may coexist as well, impacting policy support in turn.

### 5.4 Limitations and suggestions for future research

Arguably, the most important limitation of this study was not having access to data for perceptions of the need principle for pandemic‐related welfare recipients. Future studies measuring the impact of the pandemic or other disasters on welfare target groups should incorporate measurements of the need principle, as it seemed to be a salient component in the construction of traditional welfare recipients. In addition, future studies using the CARIN framework should experiment more broadly with the wording of question prompts to incorporate alternative phrasings of the principles and also to determine if ‘positively worded’ prompts receive distinct responses from ‘negatively worded’ prompts for the same principle. It is notable that the reciprocity and attitude principles were the only negatively worded principles in our study, and they simultaneously constituted ¾ of the undeserving ratings. As such, it seems worth exploring whether diversifying the wording of those particular prompts would yield different results.

One key methodological limitation of this study lies with its singular placement in time. As mentioned, this data was collected in the spring and summer of 2021, with the pandemic still salient in the mind of the public and impacting both economic and political spheres. Collecting and analysing data during this period has its costs and benefits: on the one hand, the types of inferences and systematic investigation that this data allowed for would be difficult to carry out under any other point in time. On the other hand, the generalizability of this study's conclusions—particularly going forward—will remain uncertain without further research. Therefore, future studies should focus on the stability and sustainability of the current, deserving social constructions for these two target groups.

## 6 CONCLUSION

Moving forward, public policy scholars and political scientists analysing the American welfare state should strongly consider incorporating the CARIN principles framework into their research. Linear regression models suggest that the principles of control, attitude, and identity were significantly correlated with attitudes for pandemic‐related welfare, and differences in principle ratings were significant when compared across traditional welfare and pandemic‐related welfare target groups. While typical covariates like ideology, race, and partisanship are still foundational to understanding the American public's perception of welfare, our findings provide support for the argument that the deservingness principles used in scholarly analysis around the world should be incorporated into future examinations of the welfare state in the US.

## Data Availability

The data that support the findings of this study are available from the corresponding author upon reasonable request.

## Endnote

^1^
The hypotheses for this study were pre‐registered at the Open Science Framework and can be accessed at https://osf.io/dhyf7. As they are presented in the following section, the Hypotheses may have been edited for clarity, but all substantive content remains the same. See H2.3 (the reciprocity principle) was added after being overlooked in the pre‐registration. Data for the need principle rating of pandemic‐related welfare recipients was not available for this survey, so no hypothesis is presented for that instance.

## APPENDIX A

**TABLE A1:** Comparing correlations across treatments

	Correlations for pandemic welfare principles				Correlations for traditional welfare principles			
	Control principle	Attitude principle	Reciprocity principle	Identity principle	Control principle	Attitude principle	Reciprocity principle	Identity principle
Control principle								
Attitude principle	0.13***				0.13***			
Reciprocity principle	0.07	0.60***			0.09**	0.55***		
Identity principle	0.25***	−0.16***	−0.21***		0.43***	−0.14***	−0.10**	
Need principle	0.52***	0.13***	0.03	0.21***	0.64***	0.14***	0.16***	0.37***

****p* < 0.001; ***p* < 0.01.
